# Genetic dissection of maize disease resistance and its applications in molecular breeding

**DOI:** 10.1007/s11032-021-01219-y

**Published:** 2021-05-15

**Authors:** Mang Zhu, Lixiu Tong, Mingliang Xu, Tao Zhong

**Affiliations:** State Key Laboratory of Plant Physiology and Biochemistry/College of Agronomy and Biotechnology/National Maize Improvement Center/Center for Crop Functional Genomics and Molecular Breeding, China Agricultural University, 2 West Yuanmingyuan Road, Beijing, 100193 People’s Republic of China

**Keywords:** Maize, Disease resistance, Marker-assisted selection, Genome editing, Mang Zhu and Lixiu Tong contributed equally to this manuscript.

## Abstract

Disease resistance is essential for reliable maize production. In a long-term tug-of-war between maize and its pathogenic microbes, naturally occurring resistance genes gradually accumulate and play a key role in protecting maize from various destructive diseases. Recently, significant progress has been made in deciphering the genetic basis of disease resistance in maize. Enhancing disease resistance can now be explored at the molecular level, from marker-assisted selection to genomic selection, transgenesis technique, and genome editing. In view of the continuing accumulation of cloned resistance genes and in-depth understanding of their resistance mechanisms, coupled with rapid progress of biotechnology, it is expected that the large-scale commercial application of molecular breeding of resistant maize varieties will soon become a reality.

## Introduction

Maize (*Zea mays* L.) is one of the most important crops for food, feed, and fuel production worldwide. The global demand for maize continues to grow because of the increasing need for feed and industrial raw materials. In recent years, due to the ever-shrinking genetic diversity of maize varieties, continuous cropping, returning straw to the field, high-fertility management, high-density planting, and extreme climatic events, maize diseases are becoming more and more serious (Duan et al. 2019; Sun et al. 2020). Annual yield losses caused by maize diseases (excluding viral diseases) are estimated to account for 4–14% of the global harvest (https://portal.nifa.usda.gov/web/crisprojectpages/1008502-genetic-architecture-of-disease-resistance-in-maize.html). Since the arable land area of maize will not increase significantly, it will be crucial to ensure stable maize yields and high kernel quality by reducing disease severity in this crop.

In the long history of maize cultivation, disease epidemics in maize is changing dynamically, as some diseases increase or decrease in importance relative to other diseases. Disease prevalence in maize is closely correlated with pathogen resources, cultivated varieties, weather conditions, farming systems, and agricultural ecology (Yang et al. 2017a). The outbreak of southern corn leaf blight (SCLB) in the USA in 1970 was due to the wide deployment of susceptible cultivars with T-type male-sterile cytoplasm (cms-T), which suffered from the new *Bipolaris maydis* race T (Ullstrup 1972). The recent occurrence of northern corn leaf blight (NCLB) in North China was due to the widespread cultivation of the susceptible variety XianYu335 (Pu 2013). The high temperature and humidity in the Huang-Huai-Hai plain are conducive to the outbreak of stalk rot and ear rot diseases (Duan et al. 2019). In mechanical harvesting, maize plants are left in the field for a longer period of time for dehydration, which will undoubtedly increase the severity of stalk/ear rot diseases. Moreover, the failure to remove rotted ears during mechanical harvesting further reduces kernel quality (Silva et al. 2017; Holland et al. 2020).

The plant immune response is a highly complex, tightly regulated, multi-layered process that can be roughly divided into qualitative disease resistance and quantitative disease resistance (QDR) (Poland et al. 2009; Kou and Wang 2010). Resistance genes (*R*-gene) underlying qualitative resistance tend to provide complete or near-complete resistance and are therefore also known as major genes (Nelson et al. 2018). QDR confers an incomplete or partial resistance and is controlled by multiple small-effect genes (Niks et al. 2015). Although single *R*-genes are often non-durable, when used in combination with QDR genes, they can effectively promote crop protection against pathogens (Palloix et al. 2009). Therefore, combining multiple *R*-genes and/or QDRs into a single genome is the optimal choice for breeding varieties with strong and durable disease resistance.

As early as 1992, a major gene *Hm1* was isolated in maize by transposon-tagging method, which confers resistance to *Cochliobolus carbonum* race 1 (Johal and Briggs 1992). Notably, *Hm1* is also the first resistance gene identified in any plant species. With the same transposon-tagging approach, another major gene *Rp1-D* was isolated in 1999, which confers resistance against common leaf rust (Collins et al. 1999). During the maize growth period, however, the most devastating diseases are caused by necrotrophic or hemibiotrophic pathogens, and the resistance to such diseases mainly depends on QDR genes (Yang et al. 2017a). It turns out that cloning of the QDR gene is much more difficult than the major gene. It is not until recently that there have been reports of successful cloning of QDR genes (Zuo et al. 2015; Hurni et al. 2015; Yang et al. 2017b; Wang et al. 2017; Liu et al. 2017; Leng et al. 2017; Li et al. 2019; Ye et al. 2019; Yang et al. 2021; Liu et al. 2020a).

In this review, we summarize recent advances in functional genomics on maize disease resistance, describe the current works on molecular breeding, and predict the potential development in the future. Several similar reviews may help to better understand the maize disease resistance (Poland et al. 2009; Kou and Wang 2010; St Clair 2010; Zhang et al. 2013; Niks et al. 2015; Krattinger and Keller 2016; Ali and Yan 2012; Yang et al. 2017a).

## Functional genomics of disease resistance in maize

### Inheritance of resistance to fungal diseases in maize

Most maize diseases are caused by pathogenic fungi (Table 1). These diseases cause significant economic losses due to reduced yield/quality and the increasing input cost for disease control. Foliar disease, smut, and stem/ear rot are among the most serious fungal diseases of maize (Azra and Hussain 2019).

**Table 1:** Introduction, characteristics, and inheritance of major diseases in maize

Disease	Pathogen	Pathogen type	Nutritional typea	Location of disease	Distribution	Resistance geneb
Northern corn leaf blight (NCLB)	*Exerohillum turcicum* (Teleomorph *Setosphaeria turcica*)	Fungus	Hemibiotrophic	Mainly on the leaves	Worldwide	*ZmWAK-RLK1* (Hurni et al. 2015; Yang et al. 2021), *remorin* (*ZmREM6.3*) (Jamann et al. 2016)
Southern corn leaf blight (SCLB)	*Bipolaris maydis* (Teleomorph *Cochliobolus heterostrophus*)	Fungus	Necrotrophic	Mainly on the leaves	Worldwide	*ZmCCoAOMT2* (Yang et al. 2017b), *rhm1* (Zhao et al. 2012b)
Banded leaf and sheath blight (BLSB)	*Rhizoctonia solani* (Teleomorph *Thanatephorus cucumeris*)	Fungus	Necrotrophic	Leaf and sheath	China, south, and southeast Asia	*ZmFBL41* (Li et al. 2019)
Gray leaf spot (GLS)	*Cercospora zeina*	Fungus	Necrotrophic	Mainly on the leaves	China (southwest), USA (east), South Africa	
	*Cercospora zeae-maydis*				China (northeast), USA, northern South America, Sub-Saharan Africa	*ZmCCoAOMT2* (Yang et al. 2017b)
Curvularia leaf spot (CLS)	*Curvularia lunata* (Teleomorph *Cochliobolus lunata*)	Fungus	Hemibiotrophic	Mainly on the leaves	Worldwide	
Common rust	*Puccinia sorghi*	Fungus	Biotrophic	Leaf and sheath	China (north), Africa, North America, Europe, Australia, New Zealand	*Rp1-D* (Collins et al. 1999)
Southern rust	*Puccinia polysora*	Fungus	Biotrophic	Especially on leaves	China (southwest), Americas, Africa, Asia, Australia	*ZmREM1.3* (Wang et al. 2019b)
Head smut	*Sporisorium reilianum*	Fungus	Biotrophic	Tassel and ear	Worldwide	*ZmWAK* (Zuo et al. 2015)
Common smut	*Ustilago maydis*	Fungus	Biotrophic	All aboveground organs	Worldwide	
*Gibberella* ear rot	*Fusarium graminearum* (Teleomorph *Gibberella zeae*)	Fungus	Necrotrophic	Ear	China (north), southern Brazil	*ZmAuxRP1* (Ye et al. 2019)
*Fusarium* ear rot	*Fusarium verticillioides*		Biotrophic		Worldwide	*ZmLOX3* (Gao et al. 2009), *ZmLOX12* (Christensen et al. 2014)
*Diplodia* ear rot	*Stenocarpella maydis*		Necrotrophic		USA, Kenya, New Zealand, South Africa	
*Aspergilus* ear rot	*Aspergillus flavus*		Necrotrophic		Worldwide	*ZmLOX3* (Gao et al. 2009)
*Gibberella* stalk rot	*Fusarium graminearum* (Teleomorph *Gibberella zeae*)	Fungus	Hemibiotrophic	Stalk and sheath	Worldwide	*ZmCCT* (Wang et al. 2017), *ZmAuxRP1* (Ye et al. 2019)
*Pythium* stalk rot	*Pythium aphanidermatum* and *Pythium inflatum*		Necrotrophic		Worldwide	
*Fusarium* stalk rot	*Fusarium verticillioides* and several other *Fusarium* species		Necrotrophic		USA, Canada, Mexico, India	
*Anthracnose* stalk rot	*Colletotrichum graminicola* (teleomorph *Glomerella graminicola*)		Hemibiotrophic		Worldwide	*Rcg1* (Jung et al. 1994; Frey et al. 2011)
Bacterial stalk rot	*Dickeya zeae* (Syns. *Erwinia chrysanthemi pv. Zeae*)	Bacterium	Gram-negative	Stalk and sheath	Worldwide	
Goss’s bacterial wilt and blight	*Clavibacter nebraskensis*	Bacterium	Gram-positive	Leaves and stalks	USA, Canada, Mexico, Brazil	
Maize rough dwarf disease (MRDD)	Rice black-streaked dwarf virus	Virus	Double-stranded RNA virus	Whole plant	Worldwide	*ZmGDIα-hel* (Liu et al. 2020)
Maize dwarf mosaic disease (MDMD)	Maize dwarf mosaic virus, sugarcane mosaic virus	Virus	Single-stranded RNA virus	Whole plant	Worldwide	*ZmTrxh* (Liu et al. 2017), *ZmABP1* (Leng et al. 2017)
Maize lethal necrosis (MLN)	Maize chlorotic mottle virus (in combination with one of several viruses from the *Potyviridae*)	Virus	Single-stranded RNA virus	Whole plant	Sub-Saharan East Africa, Southeast Asia, South America	

^a^In this list, the nutritional type is indicated for fungi, and the type of bacteria/virus is indicated for bacteria/virus

^b^This list contains cloned or implicated disease resistance genes in maize

**Fig. 1:**
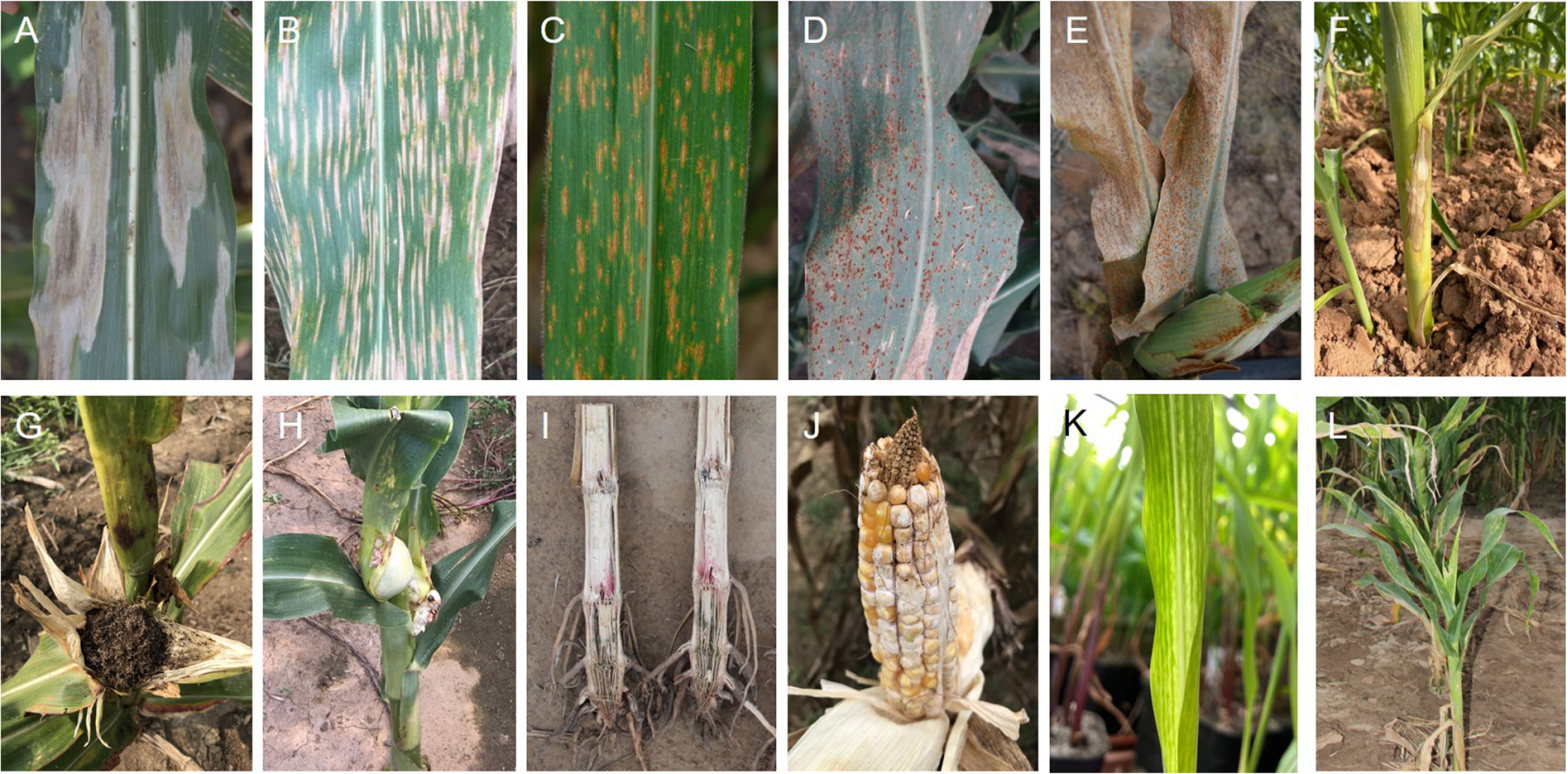
The phenotypes of major maize diseases. **a N**CLB mainly damages leaves and forms local lesions that progress until necrosis occurs. Lesions may coalesce, blighting the entire leaf. **b** GLS begins as small, regular, elongated necrotic spots. Lesions grow parallel to the veins. The growth is limited by adjacent veins, so the final lesion shape is rectangular. **c** SCLB mainly infects maize leaves. Lesions are initially small and diamond shaped, then become elongated as they mature. Under severe disease pressure, lesions may coalesce, blighting the entire leaf. **d** Common rust has small, powdery pustules over both surfaces of the leaves. Pustules are brown in early stages of infection; later, the epidermis ruptures and the lesions turn black as the plant matures. **e** Southern rust has small circular, pinhead-shaped pustules. Pustules are smaller, lighter in color, and more circular than those of common rust. Southern rust also presents on both leaf surfaces. **f** BLSB develops on leaves, sheaths, and husks. The symptoms are characteristic concentric spots that cover large areas of infected leaves and husks. **g** Head smut spreads systemically into the ear and tassel. The most conspicuous symptom is masses of black spores in the individual male florets and the ear. **h** Common smut is easily identified by white tumor-like galls which can develop in all aerial organs of maize. **i** *Gibberella* stalk rot may look similar to *Fusarium* stalk rot. Symptoms of stalk rot include rotting of the roots, crown, and lower internodes. Corn infected with *Gibberella* has pink to reddish coloration of the pith and vascular strands. **j** *Fusarium* ear rot is the most common fungal disease found on corn ears. Mold may be white, pink, or salmon-colored. Infected kernels may turn tan or brown. **k** SCMV infection causes characteristic chlorosis, here shown in a greenhouse-grown seedling. The new leaves of susceptible plants show yellow and green stripes. **l** MRDD-infected plants are usually dwarfed and severely stunted, with dark-green leaves, shortened internodes, and waxy enations on the abaxial surfaces of upper leaves

Foliar fungal diseases of cereals are usually associated with reduced photosynthetic area, chlorosis, and premature leaf senescence (Fig. 1a–e), which result in incomplete grain filling and reduced grain yields (Zheng et al. 2018). A recent global survey highlighted several foliar fungal diseases that significantly reduce maize yields in Africa, Asia, and the Americas (Savary et al. 2019).

Northern corn leaf blight (NCLB) (Fig. 1a) causes the yield loss of > 1% globally (Savary et al. 2019). In the northern USA, NCLB was the most prominent corn disease in 2015 (Mueller et al. 2016). In Jilin Province of China, the NCLB outbreak in 2012 caused a substantial yield loss (Liu et al. 2013). Qualitative resistance conveyed by *Ht* genes results in distinct phenotypes in response to infection by avirulent races of *Exserohilum turcicum*. The *Ht1* gene, identified from the popcorn cultivar Ladyfinger and the field corn inbred line GE440, confers a chlorotic-lesion reaction that reduces sporulation and lesion size (Hooker 1963). The *Ht2* and *Ht3* genes also confer chlorotic-lesion-mediated resistance (Hooker 1977; Hurni et al. 2015). The *Htn1* locus was originally introgressed into modern maize cultivars from Mexican landrace Pepitilla in the 1970s (Gevers 1975). *ZmWAK-RLK1* is the causal gene at *Htn1*, which encodes an unusual innate immunity receptor with an extracellular wall-associated kinase domain (Hurni et al. 2015), and the fungal resistance correlates with reduced benzoxazinoid content (Yang et al. 2019b). The latest research showed that *Ht2* and *Ht3* are identical and allelic to *Htn1*. The difference between the ZmWAK-RLK1 variants encoded by *Htn1* and *Ht2/Ht3* lies in multiple amino acid polymorphisms, which particularly affect the putative extracellular domain (Yang et al. 2021). The recessive gene *ht4*, identified in a line derived from the maize synthetic BS19, confers a chlorotic halo reaction to infection by *E. turcicum* (Carson 1995). Loci affecting quantitative resistance to NCLB have been mapped on all 10 maize chromosomes (Welz and Geiger 2000). *ZmREM6.3*, the causal gene of *qNLB1.02*_*B73*_, was identified by combing fine mapping, expression analysis, and mutant evaluations. *ZmREM6.3* appears to have a specific effect on NCLB symptom development (Jamann et al. 2016).

Gray leaf spot (GLS) (Fig. 1b) is the second most serious foliar disease of maize worldwide (Savary et al. 2019). GLS resistance is a typical quantitative trait controlled by multiple resistance QTLs (Menkir and Ayodele 2005). *ZmCCoAOMT2* was confirmed to be the causal gene at QTL *qMdr*_*9.02*_ conferring resistance to *Cercospora zeae-maydis* GLS. This gene encodes caffeoyl-CoA O-methyltransferase, an enzyme involved in the phenylpropanoid pathway and lignin production (Yang et al. 2017b). Another *C. zeae-maydis* GLS resistance QTL, *Qgls8*, was mapped to a ~ 130-kb region on chromosome 8 (Zhang et al. 2017b). Two major resistance QTLs against *Cercospora zeina* GLS, *qRgls1* and *qRgls2*, were identified and fine-mapped to 1.4-Mb and 1-Mb regions on chromosomes 8 and 5, respectively (Zhang et al. 2012b; Xu et al. 2014). To date, more than 100 QTLs for GLS resistance have been detected (Du et al. 2020).

Southern corn leaf blight (SCLB) (Fig. 1c), once a major threat to global maize production, has declined to a relatively low level due to the use of resistant cultivars. A major recessive SCLB resistance locus, *rhm1*, was mapped to an 8.56-kb region on chromosome 6 with only one candidate gene, encoding the lysine histidine transporter 1 (LHT1) (Zhao et al. 2012b). To date, many QTL analyses of maize resistance to SCLB have been conducted, revealing numerous resistance QTLs that primarily exhibit additive or partially dominant or epistatic effects (Carson et al. 2004; Balint-Kurti and Carson 2006; Balint-Kurti et al. 2007; Balint-Kurti et al. 2008; Zwonitzer et al. 2009; Kaur et al. 2019). *ZmCCoAOMT2*, the causal gene of the QTL *qMdr*_*9.02*_, also confers quantitative resistance to SCLB (Yang et al. 2017b). Differences in *ZmCCoAOMT2*-mediated SCLB resistance are attributed to allelic variations at both the gene expression and amino acid sequence levels, which lead to differences in the levels of metabolites (e.g., lignin) in the phenylpropanoid pathway and programmed cell death (Yang et al. 2017b).

Common rust (Fig. 1d) is an important foliar disease that is widely distributed in tropical, subtropical, temperate, and highland areas (Vivek et al. 2009; Wright et al. 2014), and causes up to 49% yield losses in susceptible genotypes (Groth et al. 1983). The *rp1* complex, a cluster of resistance genes, is located on the short arm of chromosome 10 (Hulbert 1997). Sixteen different genes were identified in the *rp1* cluster by examining their responses to an extensive collection of rust biotypes, and fourteen of which were given the *Rp1* designation (*Rp1-A* to *Rp1-N*) (Hooker 1969; Hulbert 1997). Among them, *Rp1-D*, encoding a typical resistance protein with nucleotide-binding and leucine-rich repeat (NB-LRR) domains, confers race-specific resistance to the disease (Collins et al. 1999). Since many *Puccinia sorghi* races that are virulent on *Rp1-D* have been found throughout North America (Pataky and Tracy 1999; Pate et al. 2000; Pataky et al. 2000), it is important to combine multiple *R*-genes with QDRs to generate maize varieties with durable resistance to common rust (Yang et al. 2017a). With genome-wide association studies (GWAS), 25 resistance QTLs were identified and distributed on chromosomes 1, 3, 5, 6, 8, and 10 (Zheng et al. 2018).

Southern rust (Fig. 1e) is generally more harmful to corn than common rust due to its ability to develop and spread rapidly under favorable conditions. To date, at least 18 race-specific resistance genes have been identified, and most have been widely used in commercial maize varieties, such as *Rpp1-11* (Storey and Howland 1957; Ullstrup 1965; Brewbaker et al. 2011), *Rpp25* (Zhao et al. 2013), *RppQ* (Chen et al. 2004; Zhou et al. 2007), *RppD* (Zhang et al. 2010), *RppC* (Yao et al. 2013), *RppS313* (Wang et al. 2019a), *RppS* (Wu et al. 2015), and *RppCML496* (Lv et al. 2020). Like other plant pathogenic microbes, *Puccinia polysora* is notorious for its rapid mutation to overcome maize resistance. For example, *Rpp9* once provided effective resistance to SCR in the southern USA, but it has since been overcome by a new race of *P. polysora* (Brewbaker et al. 2011). The resistance QTLs have been identified and mapped on chromosomes 3 and 4 (Holland et al. 1998); 3, 4, and 9 (Jiang et al. 1999); 4, 8, 9, and 10 (Jines et al. 2007); 6 (Brewbaker et al. 2011); and 1, 2, 5, 6, 9, and 10 (Wanlayaporn et al. 2013). Plant-specific remorins are important for plant responses to microbial infections and plant signaling processes. Overexpressing the remorin gene *ZmREM1.3* enhanced resistance to southern rust in maize (Wang et al. 2019c).

Banded leaf and sheath blight (BLSB) (Fig. 1f) is a widespread soil-borne fungal disease of both maize and rice in South and Southeast Asia (Zhao et al. 2006; Chen et al. 2013; Li et al. 2019). The F-box gene *ZmFBL41* was identified as a causal gene conferring quantitative resistance to BLSB (Li et al. 2019). The activity of *ZmFBL41* was evaluated in the transposon-insertion line *zmfbl41* selected from the maize UniformMu resource. The *zmfbl41* line exhibited weaker disease symptoms than the wild type (W22) following *Rhizoctonia solani* infection. Two amino acid substitutions in ZmFBL41 prevented its interaction with ZmCAD (the final enzyme in the monolignol biosynthetic pathway). This resulted in inhibited ZmCAD degradation, leading to lignin accumulation and limiting lesion expansion (Li et al. 2019).

Head smut (Fig. 1g) and common smut (Fig. 1h) are both soil-borne diseases of maize and pose serious threats to maize production. Many head smut resistance QTLs have been identified across all 10 chromosomes (Lübberstedt et al. 1999; Chen et al. 2008; Li et al. 2015). A major dominant QTL *qHSR1* on the long arm of chromosome 2 reduced the disease incidence by ~ 25% (Chen et al. 2008). *ZmWAK* is the causal resistance gene at *qHSR1* and encodes a cell wall–associated kinase (WAK), composing of a cytoplasmic serine/threonine kinase domain, a calcium-binding epidermal growth factor (EGF_CA) domain, and an extracellular galacturonan-binding (GUB) domain (Zuo et al. 2015). ZmWAK spans the plasma membrane and functions as a receptor-like kinase that may perceive and transduce extracellular signals. *ZmWAK* is highly expressed in the mesocotyls of maize seedlings, where it represses the growth of hyphae towards aboveground plant tissues, resulting in a significant decrease in the pathogen amount in floral organs, thereby reducing the disease severity (Zuo et al. 2015). Common smut, caused by *Ustilago maydis*, can be easily identified by the formation of tumor-like galls in all aerial organs of maize plants, which results in stunted growth and yield losses (Martínez-Espinoza et al. 2002; Tanaka et al. 2020). In recent years, great progress has been made in the study of the pathogenic mechanism of *U. maydis* and its interaction with plants (Ma et al. 2018; Tanaka et al. 2020; Zuo et al. 2019). The disease resistance loci are distributed on all 10 maize chromosomes; however, none of them have been identified (Pataky 1995; Lübberstedt et al. 1998; Ding et al. 2008).

With the development of agricultural mechanization, stalk rot (Fig. 1i) and ear rot (Fig. 1j) have attracted much attention, and prompted genetic studies on them. Two QTLs, the major *qRfg1* and the minor *qRfg2*, were identified in the resistant inbred line 1145 (Yang et al. 2010; Zhang et al. 2012a). The *ZmCCT* gene containing a CCT domain is the causal gene at *qRfg1* (Wang et al. 2017). The insertion or deletion of a CACTA-like transposon in the *ZmCCT* promoter causes differential histone modification and DNA methylation to regulate maize resistance to stalk rot (Wang et al. 2017). Without the transposon insertion, *ZmCCT* is in the “primed” state, allowing plants to respond quickly to pathogen challenge and mount defense responses. By contrast, *ZmCCT* with the transposon insertion is in the “silent” state, eliciting little or no defense response to pathogen invasion (Wang et al. 2017). *ZmAuxRP1* is the causal gene at the minor QTL, *qRfg2*, that responds quickly to pathogen challenge with a rapid yet transient reduction in its expression, leading to arrested root growth but enhanced resistance to *Gibberella* stalk rot (Ye et al. 2019). ZmAuxRP1 promotes the biosynthesis of indole-3-acetic acid (IAA), while suppressing the formation of benzoxazinoid defense compounds (BXs). The concerted interplay between IAA and BXs helps maintain the growth-defense balance in a timely and efficient manner to optimize plant fitness (Ye et al. 2019). Interestingly, *ZmAuxRP1* increases the resistance to *Fusarium* ear rot as well, suggesting that the same mechanism is used for resistance to both stalk rot and ear rot (Ye et al. 2019).

*Rcg1* is a major QTL associated with resistance to *Anthracnose* stalk rot (ASR) caused by the fungus *Colletotrichum graminicola* (Jung et al. 1994). *Rcg1* was identified in the inbred line MP305 by fine mapping, followed by mutant analysis. *Rcg1* harbors an NB-LRR disease resistance gene that delays the occurrence of *Anthracnose* stalk rot, causing the disease to have little impact on plant yield (Frey et al. 2011).

LOX (lipoxygenase) genes are thought to be involved in plant susceptibility to fungal invasion and mycotoxin production (Christensen et al. 2014; Maschietto et al. 2015). Maize mutants with a defect in the 9-LOX gene *ZmLOX3* show reduced levels of several 9-LOX-derived fatty acid hydroperoxides. The kernels of *lox3* mutants show greatly reduced ear rot symptoms, including drastically reduced conidiation of *F. verticillioides* and reduced production of the mycotoxin fumonisin B1 (Gao et al. 2007; Gao et al. 2009). By contrast, infection by *F. verticillioides* is suppressed by the maize 9-LOX gene *ZmLOX12* (Christensen et al. 2014). These observations suggest that a specific plant 9-LOX isoform is required for fungal pathogenesis, including disease development and spore and mycotoxin production (Lanubile et al. 2017).

### Inheritance of resistance to viral diseases in maize

At least ten viruses cause significant agronomic losses in maize globally (Table 1) (White 1999). The incidence and severity of viral diseases are increasing, and new viral diseases continue to emerge. Maize dwarf mosaic disease (MDMD) is prevalent worldwide, especially in the USA, Europe, and the Huang-Huai-Hai plain in China. This disease seriously affects the yield and quality of maize (Fuchs and Gruntzig 1995). Maize lethal necrosis (MLN), a complex viral disease, is emerging as a serious threat to maize production (Boddupalli et al. 2020). MLN is caused by maize chlorotic mottle virus (MCMV; genus *Machlomovirus* in the Tombusviridae) in combination with one of several viruses from the Potyviridae, such as sugarcane mosaic virus (SCMV), maize dwarf mosaic virus (MDMV), Johnsongrass mosaic virus (JMV), and wheat streak mosaic virus (WSMV) (Redinbaugh and Stewart 2018; Boddupalli et al. 2020). MLN causes irreversible damage that kills maize plants before they reach maturity (Yang et al. 2017a). During 2012–2013, the estimated maize yield losses due to MLN were 23–100% in affected counties of Kenya (De Groote et al. 2016; Batchelor et al. 2020). Maize rough dwarf disease (MRDD) (Fig. 1l) poses a grave threat to maize production worldwide (Dovas et al. 2004; Achon et al. 2015). MRDD is caused by viruses in the *Fijivirus* genus in the Reoviridae family (Zhang et al. 2001; Liu et al. 2020a). In China, outbreaks of MRDD mainly occur in the Huang-Huai-Hai plain (Chen et al. 2015; Xu et al. 2020). Yield losses caused by MRDD range from 20 to 30% to as high as 100% in severely infected fields (Xu et al. 2020).

To date, only three viral disease resistance genes have been identified and validated, including *ZmTrxh* (Liu et al. 2017) and *ZmABP1* (Leng et al. 2017) against SCMV, and *ZmGDIα* against MRDD (Liu et al. 2020a). *ZmTrxh* and *ZmABP1* are the causal genes of the major QTLs *Scmv1* and *Scmv2*, respectively (Xia et al. 1999; Xu et al. 1999), which function epistatically to confer complete resistance to SCMV (Xing et al. 2006). *ZmTrxh* encodes an atypical h-type thioredoxin, and its expression level is closely correlated with SCMV resistance (Tao et al. 2013a; Liu et al. 2017). ZmTrxh is dispersed in the cytoplasm to repress SCMV accumulation without eliciting salicylic acid- and/or jasmonic acid-mediated defense responses (Liu et al. 2017). *ZmABP1* encodes an auxin-binding protein, and its expression level is closely associated with disease resistance, indicating that *ZmABP1 cis*-regulatory elements play a key role in SCMV resistance (Leng et al. 2017). ZmABP1 mainly functions during later stages of viral infection and thus adds a second tier of resistance to the immediate response mediated by ZmTrxh (Leng et al. 2017).

The major quantitative QTL *qMrdd1* is proved to be associated with the *ZmGDIα* locus, which provides maize with recessive resistance to rough dwarf disease (MRDD) (Tao et al. 2013b; Liu et al. 2020a). *ZmGDIα* encodes a Rab GDP dissociation inhibitor alpha (RabGDIα), which is required for vesicle trafficking. The wild-type *ZmGDIα* is the dominant susceptible allele, and its splicing mutant *ZmGDIα-hel* is the recessive resistant allele. *ZmGDIα-hel* was generated when a *helitron* transposon inserted into its intron 10, inducing alternative splicing that replaces the wild-type exon 10 with a *helitron*-derived exon 10. *ZmGDIα-hel* reduces the disease severity index of MRDD by ~ 30% (Liu et al. 2020a). The viral protein P7-1 binds tightly to exon 10 and the C-terminal region of the wild-type ZmGDIα to recruit it for viral infection. The *helitron*-derived exon 10 weakens the binding of P7-1 to ZmGDIα-hel, resulting in quantitative resistance to MRDD (Liu et al. 2020a).

### Inheritance of resistance to bacterial diseases in maize

Under favorable environmental conditions, such as protected cultivation, bacterial pathogens can cause tremendous crop losses (Table 1). Since its discovery in 1969, Goss’s bacterial wilt and leaf blight has emerged as an important disease of maize that causes more than 40% yield losses in susceptible maize hybrids (Carson 1991). In recent years, this disease has re-emerged and spread throughout all major corn-growing regions in the USA and Canada (Soliman et al. 2018) and caused 12.7 million tons of yield losses in maize between 2012 and 2015 (Mueller et al. 2016). Linkage mapping using three recombinant inbred line populations identified 19 QTLs (Singh et al. 2016). The effect size of each QTL was small, and none contributed > 6% of the total phenotypic variation (Singh et al. 2016).

Bacterial stalk rot, caused by *Dickeya zeae*, is an economically important disease that reduces crops yield by 21 to 98.8% (Kumar et al. 2017). This disease occurs in America, Canada, India, and Africa and is a major disease in tropical and subtropical maize planting areas. There are many other bacterial diseases in maize, such as bacterial leaf streak of corn (caused by *Pseudomonas andropogonis*) (Vidaver and Carlson 1978) and bacterial wilt of corn (caused by *Pantoea stewartii*) (EPPO 2006). Due to increasing global temperature and humidity, bacterial diseases pose a serious threat to the security of maize production. Unfortunately, there are few genetic studies about bacterial diseases in maize, so it is necessary to screen for various resistance sources and pay more attention to dig the resistance genes.

### Inheritance of resistance to oomycete diseases in maize

Downy mildew (DM) diseases are caused by various fungal species in several genera of Oomycetes. This major group of diseases affects many crops, including maize and sorghum. With a recombinant inbred line (RIL) from the cross between B73 (susceptible) and Ki11 (resistant), seven QTLs were identified for three DM strains, located on chromosomes 2, 3, 6, and 9. The major QTL on chromosome 2 could explain 12.95% of the total phenotypic variation (Kim et al. 2020).

*Pythium* produces a white, rapidly growing mycelium, which can infect maize and cause a variety of diseases (Agrios 2005). *Pythium* stalk rot, caused by *Pythium aphanidermatum* and *Pythium inflatum*, is a serious disease that impairs maize production (Duan et al. 2019). Two independently inherited dominant genes, *RpiQI319-1* and *RpiQI319-2*, confer resistance of *Pythium* stalk rot in maize (Song et al. 2015). Infection with *P. aphanidermatum* can also cause root rot, seedling blight, and seed rot (Wang and Duan 2020).

## Molecular breeding of disease-resistant maize

**Fig. 2:**
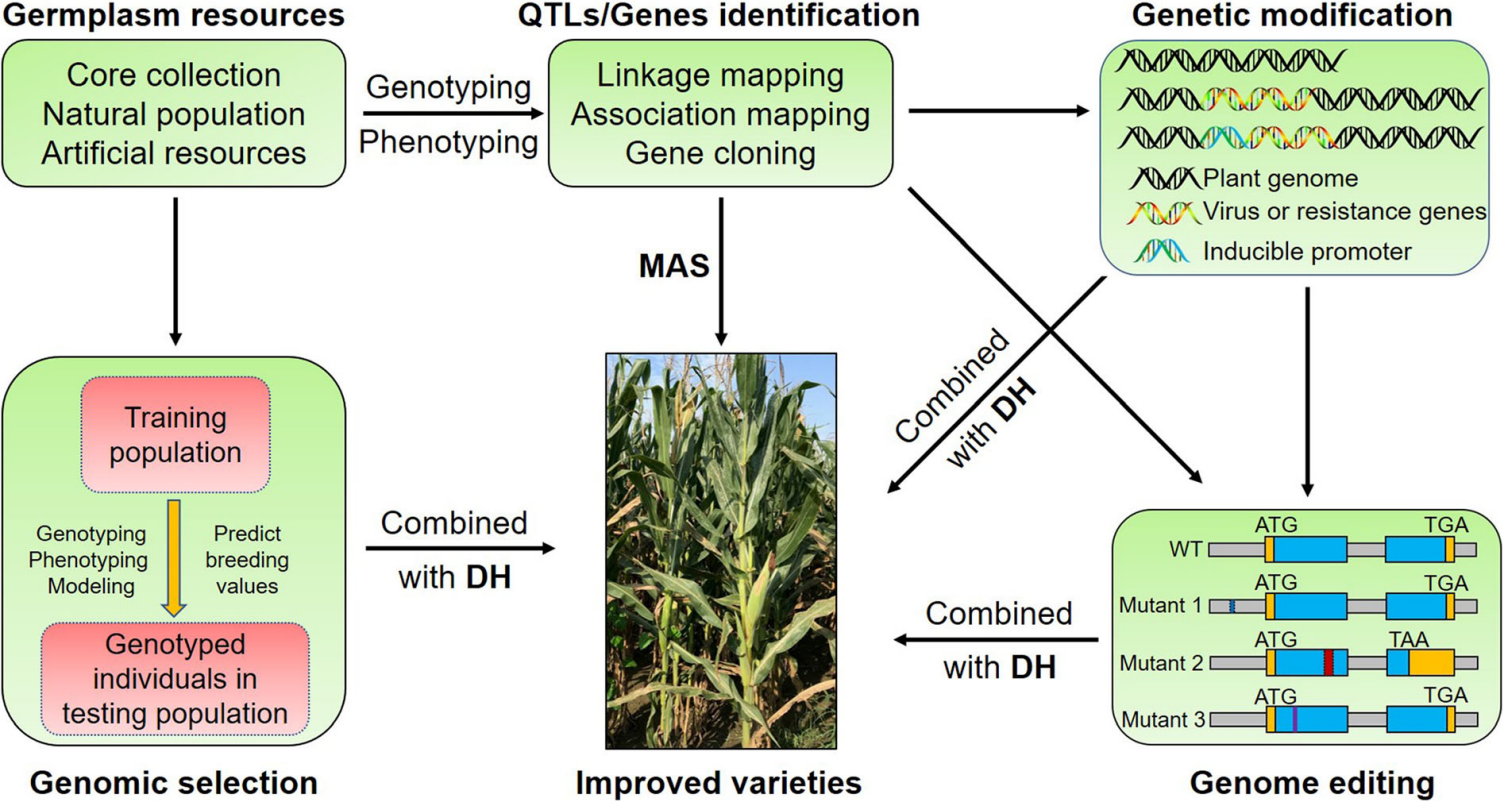
Scheme for molecular breeding of disease-resistant maize. The collection of various excellent germplasm resources can provide sources for cloning and identification of disease resistance genes. The disease resistance genes can be used for MAS and transgenic disease resistance breeding. GS can speed up resistance breeding programs in maize. Combining gene editing with DH technology can quickly generate disease-resistant materials without transgenic components. Combining various strategies is an excellent way to greatly accelerate the maize breeding process. MAS, maker-assisted selection; DH, doubled haploid; WT, wild type

Most inbred lines used in current commercial maize production are far from ideal in terms of disease resistance. For instance, very few elite inbred lines with resistance to head smut, stalk rot, and ear rot are available in China (Wang et al. 2014a; Duan et al. 2015). Several inbred lines with resistance against common rust and southern rust in China are highly susceptible to NCLB, SCLB, CLS, and GLS (Wang et al. 2014a). As known, the traditional breeding of disease-resistant varieties mainly depends on the breeder’s experiences in phenotypic selection. This is a time-consuming, inefficient process and highly dependent on environmental conditions. With the availability of elite disease resistance genes and their tagged molecular markers, the combination of traditional breeding and marker-assisted selection (MAS) has proven to be very efficient for developing elite resistant lines for maize production. Genomic selection (GS), transgenesis technique, and genome editing are all promising approaches as well. Combining these methods with doubled haploid (DH) technology could greatly accelerate the molecular breeding process in maize (Fig. 2).

### Sources of elite naturally occurring resistance genes

Natural germplasm resources, also known as genetic resources, show extensive genetic diversity in terms of disease resistance. Most disease resistance genes are present in tropical inbred lines, likely because high temperatures and high humidity favor the occurrence and maintenance of resistance genes. QTLs conferring resistance to GLS were identified in the highly resistant maize line Y32, derived from the tropical germplasm Suwan1 (Zhang et al. 2012b; Xu et al. 2014). The hybrid P78599, containing mixed ETO germplasm from South America and Suwan1 from Thailand, shows high resistance to most maize diseases. For instance, the stalk-rot disease resistance genes *ZmCCT* and *ZmAuxRP1* were isolated from P78599-derived inbred line 1145 (Wang et al. 2017; Ye et al. 2019). Teosinte, the progenitor of maize, is another important genetic resource for maize improvement, as maize has lost a great deal of genetic variation compared to teosinte due to domestication and breeding bottlenecks (Tenaillon et al. 2004). A resistance QTL derived from teosinte conferred resistance to GLS, highlighting the potential value of teosinte for maize breeding programs (Zhang et al. 2017b). Furthermore, some important resistance alleles are rare in maize germplasms. For instance, *ZmCCT* was identified solely in inbred lines bred from the P78599 hybrid (Yang et al. 2013; Wang et al. 2017; Li et al. 2017). The *ZmGDIα-hel* allele against MRDD was detected in only 36 lines among the more than 1000 lines tested (Liu et al. 2020a). Thus, it is very necessary to conduct large-scale collection and evaluation of maize germplasm before identifying importantly rare natural resistance genes and using them in resistant breeding program.

### Marker-assisted selection

MAS is a powerful tool to reduce maize diseases by using natural resistance genes. The introduction of the head smut resistance QTL *qHSR1* via marker-assisted backcrossing significantly enhanced disease resistance of 10 inbred lines (Zhao et al. 2012a). *ZmWAK*, the causal gene at *qHSR1*, improves both head smut resistance and yield-related traits (Konlasuk et al. 2015; Zuo et al. 2015). *ZmWAK* has been widely used in the head smut resistance breeding program via MAS to improve the local Chinese susceptible germplasm Tongsipingtou and to develop a number of elite inbred lines (such as Ji853R and Chang7-2R) and resistant maize varieties (e.g., Jidan558) (Zhao et al. 2012a).

*ZmCCT* and *ZmAuxRP1* are the causal factors for resistance to stalk rot (Wang et al. 2017; Ye et al. 2019). Both *ZmCCT* and *ZmAuxRP1* have pleiotropic effects: *ZmCCT* is associated with photoperiod sensitivity, and *ZmAuxRP1* is involved in root growth (Yang et al. 2013; Wang et al. 2017; Ye et al. 2019). Nine resistant *ZmCCT* haplotypes were introduced into seven elite inbred lines via MAS. The elite haplotype H5, selected from American inbred line GEMS14, exhibited enhanced resistance to stalk rot and less sensitivity to photoperiod (Li et al. 2017). Notably, inbred lines and hybrids carrying H5 also show stable stalk-rot resistance, little or no photosensitivity, and improved agronomic traits (such as yield and yield-related components). The H5 haplotype has been widely used for MAS in the stalk-rot resistance breeding programs in China and is expected to greatly alleviate the severity of stalk rot (Lanubile et al. 2017). Other stalk-rot resistance QTLs, such as *qRfg3* (Ma et al. 2017), *Rpi1* (Yang et al. 2005), and *RpiQI319-1*/*2* (Song et al. 2015), have been identified in maize that will also be useful for reducing stalk rot diseases. If a resistance gene with pleiotropic effects is selected for resistant breeding program, it is necessary to simultaneously evaluate its resistance performance and influence on other traits in multiple genetic backgrounds.

Frequently, an inbred line bearing a single resistance QTL is not enough to achieve high-resistance performance. Hence, pyramiding of various resistance genes is an effective way to reduce disease severity. The introgression of *Scmv1* and *Scmv2* into the susceptible line F7 via MAS produced a near-isogenic line (NIL) with almost complete resistance to SCMV (Xing et al. 2006). Similarly, a maize NIL containing the *qMdr9.02* locus with multiple disease resistance genes generated via MAS showed resistance to two important foliar diseases: SCLB and GLS (Yang et al. 2017b). MAS combined with phenotypic selection is a highly efficient, low-cost method that has greatly enhanced resistance breeding programs in maize (Yousef and Juvik 2001; Asea et al. 2012). However, MAS relies on the availability of markers linked to genes/dQTLs, which usually take a long time to identify by linkage or association mapping, especially for small-effect resistance QTLs. Because of this, several other strategies are currently used in disease resistance breeding programs.

### Genomic selection

Genomic selection (GS) is conducted by combining genotypic (markers) and phenotypic data in a training population to estimate the breeding values of lines that have been genotyped but not phenotyped in a testing population (Meuwissen et al. 2001). GS has been successfully used in both animal and plant breeding programs, as it substantially increases the rate of genetic gain (Meuwissen et al. 2001). The prediction accuracy of GS is influenced by many factors, including trait heritability, prediction model, population size and structure (relationship between the training and testing populations), number of markers, and genotype × environment (GE) interactions. GS uses all markers to predict the breeding value of individuals in the testing population, and thus has a greater predictive power compared to approaches that use only markers with significant effects (Massman et al. 2013). Combining two different heterotic groups in a single training set can lead to significantly more accurate prediction for both heterotic groups, and more importantly, this approach saves available resources by avoiding the need to establish a training set of sufficient size for each heterotic group (Technow et al. 2013).

Although GS in maize is currently focused on grain yield, drought tolerance, and kernel zinc and oil content, it shows promise for use in disease-resistant corn breeding programs, as the prediction accuracy for resistance to NCLB reached ~ 0.7 (Technow et al. 2013; Beyene et al. 2015; Vivek et al. 2017; Guo et al. 2020; Mageto et al. 2020; Hao et al. 2019). For MLN resistance, GS gave a promising result despite being highly influenced by the number of markers, training population size, and population relevancy (Sitonik et al. 2019; Nyaga et al. 2019). The average accuracy ranges from 0.46 to 0.86 for the MLN disease severity and 0.46 to 0.87 for the MLN area under disease progress curve (Sitonik et al. 2019). GS also showed moderate-to-high accuracy in predicting *Fusarium* ear rot resistance, in which the maximum prediction accuracy was 0.46 for *Fusarium* ear rot and 0.67 for fumonisin (Liu et al. 2020b; Kuki et al. 2020; Holland et al. 2020). The prediction accuracy could be greatly elevated if using improved training population. For instance, GS generally showed low-to-moderate prediction accuracy of 0.29 to 0.56 for GLS resistance, which could be elevated to 0.77 or even 0.84 when increasing the diversity of the training set (Kibe et al. 2020). In any case, when trying to breed a resistant hybrid to a specific disease, both parental lines should be sufficiently resistant.

### Transgenesis technique

Transgenic methods are useful for breeding disease-resistant maize. These techniques involve the direct introduction or modification of a target gene of interest using biotechnology (Christou 2013). Almost all disease resistance genes in maize function normally in the resulting transgenic lines, indicating that these techniques will be highly valuable for maize resistance breeding programs. More importantly, transgenic techniques can break the reproductive isolation between plant species to allow the introduction of resistance genes from other plant species. For instance, the expression of the durable wheat disease resistance gene *Lr34* in maize conferred resistance to common rust and NCLB (Sucher et al. 2017). Maize containing the *Rxo1* locus showed a strong hypersensitive response to a non-host bacterial pathogen (Zhao et al. 2004b; Zhao et al. 2004a). Pyramiding of different plant defense response genes and anti-apoptosis genes via genetic transformation conferred resistance to sheath blight disease and SCLB in maize (Zhu et al. 2018). In addition to resistance genes from plants, genes from fungi and viruses are also valuable in maize disease resistance breeding programs.

Functional analysis of resistance genes cloned from maize indicates that resistance performance is often closely associated with the expression of resistance gene. Thus, it is likely that disease-resistant maize could be bred by overexpressing or knocking down a gene of interest. However, the increased expression of some pleiotropic resistance genes can have negative effects on other traits. For example, in addition to conferring stalk rot resistance, overexpressing *ZmCCT* delayed flowering time in maize (Yang et al. 2013; Wang et al. 2017). The adverse effects of overexpression could be avoided by expressing a resistance gene under the control of a pathogen-inducible promoter. Although no such study has been reported in maize, this strategy has been highly successful in rice (Helliwell et al. 2013; Liu et al. 2019).

RNA interference (RNAi) induces post-transcriptional gene silencing via the expression of double-stranded RNA (dsRNA) or hairpin RNA (hpRNA). RNAi is a highly efficient method for controlling viral diseases. Expressing hpRNA derived from the capsid protein genes of MDMV and SCMV significantly enhanced maize resistance to MDMV and SCMV, respectively (Zhang et al. 2011; Gan et al. 2014). MLN, a viral disease caused by co-infection with several viruses, is destructive to maize production in Africa (Redinbaugh and Stewart 2018), suggesting that RNAi may be useful for controlling MLN.

However, transgenic approaches are not as widely used as MAS in breeding of disease-resistant maize. This is likely due to the shortage of available resistance genes and the restrictions imposed on the cultivation of genetically modified maize in many countries. Therefore, innovative transgene-free techniques have been developed that are more acceptable to disease-resistant maize breeding programs.

## Genome editing by CRISPR/CAS9

Significant progress has been made in the field of genome editing, from zinc finger nucleases (ZFNs) to transcription activator-like effector nucleases (TALENs) to clustered regularly interspaced short palindromic repeats (CRISPR)–associated protein (CRISPR/Cas) (Carroll 2014; Yin et al. 2017; Adli 2018; Gao 2021). Most genome editing technologies involve the creation of double-strand breaks (DSBs) to trigger DNA repair mechanisms (Carroll 2014; Adli 2018). DSBs are mainly repaired in one of the following two ways: error-prone non-homologous end-joining (NHEJ), which creates small insertions and/or deletions (indels), and error-free homology-directed repair (HDR), which results in the insertion or replacement of homologous DNA (Carroll 2014). The NHEJ-mediated introduction of indels can disrupt the target gene’s function if they occur in the coding region or alter the gene expression if they occur in the *cis*-regulatory region. The HDR pathway requires the use of donor homologous DNA to introduce precise insertions or substitutions (Adli 2018).

The CRISPR/Cas9 system has revolutionized the genome editing due to its simplicity, flexibility, consistency, and high efficiency and has thus become the most powerful tool for genetic analysis and crop improvement (Hua et al. 2019; Zhu et al. 2020; Gao 2021). In the past few years, the CRISPR/Cas9 system has been successfully used for plant disease control (Langner et al. 2018; Chen et al. 2019; Mao et al. 2019). Moreover, once the genome has been edited, the transgenic cassette can be eliminated by selfing or hybridization (Hua et al. 2019).

### Replacement/knock-in with dominant or partially dominant resistance genes

The major *R*-genes *Hm1* and *Rp1-D* act in a dominant manner (Johal and Briggs 1992; Collins et al. 1999). A number of QDR genes also act in a dominant or partially dominant manner, such as *ZmWAK* (Zuo et al. 2015), *Htn1* (Hurni et al. 2015), *ZmCCoAOMT2* (Yang et al. 2017b), and *ZmAuxRP1* (Ye et al. 2019). Such dominant (or partially dominant) *R* or QDR genes could be used to replace their weak or null counterparts by CRISPR/Cas9. Alternatively, these genes could be inserted into (or even stacked into) the maize genome by CRISPR/Cas9-mediated knock-in. The introduction of natural resistance genes in this manner would have many advantages, such as the lack of linkage drag, little or no fitness penalty, and stronger resistance due to the presence of multiple copies (Luo et al. 2016).

Due to the low efficiency of HDR, there are few successful examples of the replacement or knock-in of genes in plants. One of them was the improvement of drought tolerance of maize (Shi et al. 2017). Under drought-stress conditions, plants overexpressing *ARGOS8* showed reduced sensitivity to ethylene and increased grain yield. However, the abundance of endogenous *ARGOS8* transcript is relatively low in most maize inbred lines (Shi et al. 2015). The same research team used CRISPR/Cas9 technology to knock-in the *GOS2* promoter to replace the original *ARGOS8* promoter, leading to the production of plants with high levels of chimeric *ARGOS8* transcripts and enhanced drought tolerance (Shi et al. 2017). Recently, a high-frequency and selectable marker-free intra-genomic gene targeting (GT) was reported in maize, in which a heat shock–inducible Cas9 was used to simultaneously generate double-strand breaks at the target locus and release the donor template from pre-integrated T-DNA, generating up to 4.7% targeted insertion in T_0_ plants (Barone et al. 2020). This gene targeting opens up a new way to use the CRISPR-Cas9 system to repair endogenous defective alleles, a technique with great potential for improving disease resistance in maize.

### Modifying the *cis*-regulatory elements of resistance genes

Gene expression is regulated at both the transcriptional and post-transcriptional levels. The former mainly depends on regulatory elements in the promoter region, while the latter includes pre-RNA splicing, mRNA modification, mRNA transport, and mRNA degradation (Pramanik et al. 2020). *Cis*-regulatory elements are readily accessible targets for CRISPR/Cas9 (Swinnen et al. 2016). The promoter of the citrus canker susceptibility gene *CsLOB1* contains the pathogen’s effector binding element. When they were edited by CRISPR/Cas9, the resultant plants showed enhanced resistance to citrus canker (Peng et al. 2017). Similarity, mutations of the promoters of *SWEET11*, *SWEET13*, and *SWEET14* in rice conferred robust, broad-spectrum resistance to *Xanthomonas oryzae* pv. *oryzae* (Oliva et al. 2019; Xu et al. 2019).

The pleotropic gene *ZmCCT* confers quantitative resistance to *Gibberella* stalk rot and delays flowering time under long-day conditions (Yang et al. 2013; Wang et al. 2017). Thus, we reasoned that deleting the photosensitive elements in the *ZmCCT* promoter region would create an artificial allele with reduced photosensitivity but the same level of stalk rot resistance. We recently used CRISPR/Cas9 to systematically delete the photosensitive elements in the *ZmCCT* promoter to create *ZmCCT* variants with the aim to select an artificial *ZmCCT* allele to meet the requirement (unpublished data).

### Inactivation of host susceptibility factors

Host susceptibility (S) factors can be exploited by pathogenic microbes to facilitate their proliferation. Disabling these key links between plants and pathogens might provide the host with broad-spectrum, durable disease resistance (Langner et al. 2018; Zaidi et al. 2018). A classic example of the use of CRISPR/Cas9 to improve plant disease resistance involves the *S* gene *MLO*, which is conserved throughout monocots and dicots. Two teams successfully edited *MLO* in different species (wheat and tomato) through CRISPR/Cas9, and the edited *mlo* gene improved resistance to powdery mildew in both species (Wang et al. 2014b; Nekrasov et al. 2017) (Wang et al. 2014b; Nekrasov et al. 2017). Similarly, *OsERF922*, encoding the negative regulator of rice blast resistance (Liu et al. 2012), was successfully knocked out by CRISPR/Cas9, thereby increasing resistance to rice blast (Wang et al. 2016). In maize resistance to BLSB, ZmFBL41 is a negative regulator, and the transposon-insertion line *zmfbl41* improved maize resistance to BLSB (Li et al. 2019). This indicates that direct knockout of *Zmfbl41* via CRISPR/Cas9 technology can also enhance the BLSB resistance.

Since its appearance, CRISPR/Cas9 technology has been extensively exploited to meet various demands. Among them, base editing is an ideal solution for nucleotide conversion. By fusing a CRISPR-Cas9 variant with cytidine deaminase (or adenosine deaminase), base editing allows for the direct transition of C·G to T·A (or A·T to G·C) at the target site without the need of DSBs (Shimatani et al. 2017; Zong et al. 2017; Zong et al. 2018; Li et al. 2018; Chen et al. 2019; Lin et al. 2020; Gao 2021). DSB-free base editing can be used to introduce a stop codon at a specific position, thus avoiding the side effects of DSBs (Billon et al. 2017). Given that disease resistance resulting from the knockout of an *S* gene is often accompanied by fitness costs, base editing could greatly reduce changes to the target S protein, thereby minimizing fitness costs (Zaidi et al. 2018). For instance, *ZmGDIα-hel* is the recessive resistance gene against RBSDV (Liu et al. 2020a). If the key amino acids in ZmGDIα that bind to the viral P7-1 protein are identified, then base editing can be used to modify *ZmGDIα* to disrupt the interaction between ZmGDIα and P7-1, thereby generating stronger resistance *ZmGDIα* alleles.

### Combining genome editing and double-haploid technology

The removal of the CRISPR/Cas9 cassette requires several generations. Double-haploid (DH) technology is a powerful tool to promote the breeding efficiency by reducing the need for multiple generation selection (Ren et al. 2017). Combining CRISPR/Cas9 with DH technology represents an excellent way to accelerate maize breeding. Using roughly similar methods, the Haploid Induction Editing (HI-Edit) technique (Kelliher et al. 2019) and Haploid-Inducer Mediated Genome Editing (IMGE) system (Wang et al. 2019b), two different teams obtained edited haploids without the CRISPR/Cas9 cassette in a single step. Using the HI-Edit technique, 4.8–8.8% of the resulting plants were shown to lack the CRISPR/Cas9 cassette and contain the edited inducer gene *matl* (Kelliher et al. 2019). Similarly, using the IMGE system, the editing efficiency of *ZmLG1* in haploids was estimated to be ~ 4.1%, and all *zmlg1*-haploids were Cas9-free (Wang et al. 2019b).

## Perspectives

It is a daunting task to clone a QDR gene, especially a small-effect QDR gene (Yang et al. 2012). Thus far, only a few QDR genes have been cloned, and many more QDR genes remain to be identified (Mackay et al. 2009; Yang et al. 2017a). In the long run, however, it will be crucial to clone all resistance genes and understand their resistance mechanisms. This is because (1) only if resistance genes and related markers are available, can we replace susceptibility genes with resistance genes with less or no genetic drag; (2) the availability of a natural resistance gene allows for the identification of other downstream resistance-related genes in the same defense pathway; (3) all resistance genes could be modified via gene editing to create a series of artificial alleles for breeding of potentially resistant varieties.

Since most maize QDR genes only contribute a small genetic effect to help reduce disease severity (Holland 2007; Mackay et al. 2009), a lot of time and efforts are required to complete gene discovery by traditional map-based cloning strategy. In an attempt to accelerate gene discovery process, various resources must be utilized, such as diverse genetic stocks, various biological techniques, big data analysis, and bioinformatics tools. Sequential fine mapping based on recombinant-derived progeny is highly effective for narrowing down small-effect QDR loci (Yang et al. 2012; Ye et al. 2019). Online access to ever-increasing maize genome sequences is very helpful for identifying candidate resistance genes (Schnable et al. 2009; Springer et al. 2018; Sun et al. 2018; Yang et al. 2019a). Omic analysis, such as transcriptomics and metabolomics, play key roles in identifying candidate resistance genes and understanding their mechanisms (Zhang et al. 2017a; Yang et al. 2019b; Ye et al. 2019; Yao et al. 2020). Various Mu-/EMS-induced mutation libraries allow us to quickly examine the resistance performance of the candidate gene (Lu et al. 2018; Liang et al. 2019). Transgenic techniques, coupled with more powerful genome editing tools, can be used to accurately identify function of a candidate resistance gene (Christou 2013; Adli 2018).

Most sequence changes between resistant and susceptible alleles are related to transposable elements, i.e., the presence/absence variations resulted from transposon insertions, such as *ZmCCT* (Wang et al. 2017) and *ZmGDIα* (Liu et al. 2020a), or residual sequences caused by frequent transposon insertion/deletion activities, such as *ZmWAK* (Zuo et al. 2015), *ZmTrxh* (Liu et al. 2017), and *ZmABP1* (Leng et al. 2017). Given that transposable elements account for approximately 85% of the whole maize genome (Schnable et al. 2009), it is conceivable that one transposon or another will be activated by biotic stresses to create genetic variants for natural selection. Only those alleles with enhanced disease resistance and no negative effect on agronomic traits are prone to be selected and preserved in maize.

Genome editing opens up infinite possibilities to edit a target gene based on a human’s blueprint. If a resistance gene comes from the loss-of-function of a susceptibility gene, i.e., the so-called recessive resistance gene (usually found in viral resistance), the simplest way is to disrupt or delete the susceptibility gene by gene editing to create an artificial resistance allele. Alternatively, key nucleotides related to disease susceptibility need to be identified and modified to generate resistance alleles while maintaining the other functions. If a resistance gene is dominant/semi-dominant over the susceptibility gene due to the gene expression level, the *cis*-regulatory region could be modified by inserting a strongly induced promoter or increasing the copy number of resistance gene to achieve stronger resistance. On the other hand, if protein structure is essential for disease resistance, the key residues/peptides to perceive pathogen effectors should be pinpointed in an attempt to generate stronger resistance alleles. Furthermore, all genes involved in the defense pathway are the potential targets for gene editing to increase resistance.

In short, only by discovering enough resistance genes and understanding their molecular mechanisms, coupled with advanced biotechnology, can we achieve the goal of breeding super maize varieties with high disease resistance and ideal agronomic traits.

## Data Availability

Not applicable.
