# Correction to: Genetic dissection of maize disease resistance and its applications in molecular breeding

**DOI:** 10.1007/s11032-021-01250-z

**Published:** 2021-09-14

**Authors:** Mang Zhu, Lixiu Tong, Mingliang Xu, Tao Zhong

**Affiliations:** State Key Laboratory of Plant Physiology and Biochemistry/College of Agronomy and Biotechnology/National Maize Improvement Center/Center for Crop Functional Genomics and Molecular Breeding, China Agricultural University, 2 West Yuanmingyuan Road, Beijing, 100193 People’s Republic of China

**Correction to** **:** **Mol Breeding (2021) 41: 32**

**https://doi.org/10.1007/s11032-021-01219-y**

The article Genetic dissection of maize disease resistance and its applications in molecular breeding, written by Mang Zhu, Lixiu Tong, Mingliang Xu and Tao Zhong, was originally published Online First without Open Access. After publication in volume 41, issue 5, article ID 32, pages 1–22 the author decided to opt for Open Choice and to make the article an Open Access publication. Therefore, the copyright of the article has been changed to © The Author(s) 2021 and the article is forthwith distributed under the terms of the Creative Commons Attribution 4.0 International License, which permits use, sharing, adaptation, distribution and reproduction in any medium or format, as long as you give appropriate credit to the original author(s) and the source, provide a link to the Creative Commons licence, and indicate if changes were made. The images or other third party material in this article are included in the article’s Creative Commons licence, unless indicated otherwise in a credit line to the material. If material is not included in the article’s Creative Commons licence and your intended use is not permitted by statutory regulation or exceeds the permitted use, you will need to obtain permission directly from the copyright holder. To view a copy of this licence, visit http://creativecommons.org/licenses/by/4.0/.

The original article has been corrected.

